# Local modulation of steroid action: rapid control of enzymatic activity

**DOI:** 10.3389/fnins.2015.00083

**Published:** 2015-03-19

**Authors:** Thierry D. Charlier, Charlotte A. Cornil, Christine Patte-Mensah, Laurence Meyer, A. Guy Mensah-Nyagan, Jacques Balthazart

**Affiliations:** ^1^Institut de Recherche en Santé, Environnement et Travail, University of Rennes 1Rennes, France; ^2^Department of Biological Sciences, Ohio UniversityAthens, OH, USA; ^3^GIGA Neuroscience, University of LiègeLiege, Belgium; ^4^INSERM1119, University of StrasbourgStrasbourg, France

**Keywords:** aromatase, hypothalamus, 17β-estradiol, catechol-estrogens, phosphorylation, neurosteroidogenesis

## Abstract

Estrogens can induce rapid, short-lived physiological and behavioral responses, in addition to their slow, but long-term, effects at the transcriptional level. To be functionally relevant, these effects should be associated with rapid modulations of estrogens concentrations. 17β-estradiol is synthesized by the enzyme aromatase, using testosterone as a substrate, but can also be degraded into catechol-estrogens via hydroxylation by the same enzyme, leading to an increase or decrease in estrogens concentration, respectively. The first evidence that aromatase activity (AA) can be rapidly modulated came from experiments performed in Japanese quail hypothalamus homogenates. This rapid modulation is triggered by calcium-dependent phosphorylations and was confirmed in other tissues and species. The mechanisms controlling the phosphorylation status, the targeted amino acid residues and the reversibility seem to vary depending of the tissues and is discussed in this review. We currently do not know whether the phosphorylation of the same amino acid affects both aromatase and/or hydroxylase activities or whether these residues are different. These processes provide a new general mechanism by which local estrogen concentration can be rapidly altered in the brain and other tissues.

## Introduction

The local conversion of androgens into estrogens in specific areas of the central nervous system is an essential step in the activation of numerous testosterone-dependent physiological and behavioral processes, including sexual differentiation of the brain, negative feedback on the secretion of gonadotrophin releasing hormone and the activation of male sexual and aggressive behaviors (Beyer et al., 1976; MacLusky and Naftolin, 1981; Bagatell et al., 1994; Fisher et al., 1998; Honda et al., 1998; Toda et al., 2001; Rochira et al., 2006; Trainor et al., 2006). This transformation is catalyzed by the enzyme aromatase, also known as CYP 19 or estrogen synthase. In most vertebrates, central aromatase expression is restricted to specific neuronal populations mainly located in the hypothalamic/preoptic areas including the medial preoptic nucleus and in the limbic system (Roselli et al., 1985; Foidart et al., 1995a,b; Wagner and Morrell, 1997; Trainor et al., 2007; Metzdorf et al., 1999; Roselli and Resko, 2001). It should be noted that songbirds have an additional population of aromatase expressing neurons in the caudomedial nidopallium, in the telencephalon (NCM; Shen et al., 1995; Saldanha et al., 2000). Aromatase is also abundantly expressed in the whole brain of teleost fishes but only in glial cells (Forlano et al., 2001, 2006; Diotel et al., 2010). The presence of aromatase in defined brain areas allows for local increase in estrogens (17β-estradiol or E2) without affecting the periphery or surrounding brain regions. The estrogens resulting from local aromatization bind to estrogen receptors alpha or beta, trigger a conformational change and dimerization of the receptor, and lead the receptor to translocate to the nucleus. The receptor dimer then binds to a specific response element on the DNA, usually but not only, upstream of the target gene, recruits a set of transcriptional coregulators and enhances or reduces gene transcription. This modulation of transcription affects the physiology of the cell and ultimately the functioning of the organ and the entire organism. The actions of estrogens at this genomic level develop relatively slowly (several minutes but usually several hours to several days) and have long lasting effects on the physiology, from several days to a life time. However, numerous laboratories have now described estrogen-dependent physiological changes that occur much more rapidly and independently of mRNA transcription and protein synthesis. For example, an acute estrogenic treatment *in vitro* triggers the activation of numerous intracellular signaling pathways including phosphorylation of the mitogen-activated protein kinase (MAPK) and cAMP response element binding protein (CREB) and changes in intracellular calcium concentrations (Mermelstein et al., 1996; Moss et al., 1997; Kenealy et al., 2011; Roepke et al., 2005). In the brain, these modifications lead to modulations of neuronal activity (Joels, 1997; Moss et al., 1997; Rønnekleiv and Kelly, 2002; Abraham et al., 2003, 2004; Boulware et al., 2005) and can in some instance rapidly affect behavior in numerous species (Hayden-Hixson and Ferris, 1991; Remage-Healey and Bass, 2004; Dewing et al., 2007; Micevych and Mermelstein, 2008; Lord et al., 2009; Seredynski et al., 2013; see Cornil et al., 2012a for review). These observations led to the hypothesis that estrogens are not only slow acting signaling molecules, or hormones, but can also function more rapidly like classical neurotransmitters, such as glutamate or dopamine (Balthazart and Ball, 2006; Saldanha et al., 2011).

While these rapid, non-genomic, effects of estrogens are currently the focus of intense research, they raise the question of how rapid increases of estrogens can occur. Indeed, estrogens, like other steroids, cannot be stored in synaptic vesicles before rapid release due to their lipophilic nature. We therefore suggested that the fast effects of estrogens require a rapid change of local steroid concentration via rapid changes in their synthesis rate by androgen conversion (Balthazart and Ball, 2006; Cornil et al., 2006), therefore implicating changes in aromatase activity (AA). Changes in AA often reflect changes in aromatase protein concentration resulting from relatively slow transcriptional control. For example, aromatase expression in the hypothalamus in most vertebrates is controlled by sex steroids: weak aromatase expression is detected in castrated males, while testosterone replacement markedly increases aromatase transcript, protein and activity (Roselli and Resko, 1984, 1989; Schumacher and Balthazart, 1986; Fusani et al., 2001). These changes in AA generally occur after several hours or days of treatment. However, the rapid effects of E2 introduced above require mechanisms affecting the synthesis of the steroid more rapidly, suggesting posttranslational change(s) of the protein activity.

## Rapid modulation of aromatase activity

The first evidence that AA is rapidly modulated was obtained in preoptic area/hypothalamus explants from Japanese quail. A rise in extracellular K^+^ concentration or an increase of intracellular calcium concentration rapidly (5 min) and transiently reduced AA (Balthazart et al., 2001b). These results suggested that the K^+^-induced transient depolarization resulted in an increase of Ca^++^ from intracellular stores and led to a rapid reduction of AA. In agreement with this hypothesis, the inhibition of enzymatic activity was significantly hindered when the K^+^-induced depolarization was performed in a Ca^++^-free medium (Balthazart et al., 2001b). In a different model, the zebra finch telencephalon, Remage–Healey and colleagues showed that a K^+^-induced depolarization and the increase of intracellular calcium concentrations were also able to significantly reduce AA, as suggested by the rapid reduction of E2 concentrations measured by ELISA on NCM microdialysates (Remage-Healey et al., 2011). However, the increase in calcium concentrations in this model resulted from the activation of voltage-gated calcium channels, and not from a release from intracellular stores. Indeed, the use of ω–conotoxin, a specific inhibitor of N-type Ca^++^ channel, blocked the K^+^-induced decrease in E2 concentration (Remage-Healey et al., 2011).

### Glutamate

Our group investigated physiologically relevant signaling molecules that could affect the local concentrations of calcium in the brain and investigated whether the activation of glutamate receptors could lead to a rapid modulation of AA in male quail preoptic/hypothalamic explants. We found that the glutamate agonists AMPA [2-amino-3-(5-methyl-3-oxo-1,2- oxazol-4-yl) propanoic acid], kainate and, to a some extent, NMDA (N-methyl-D-aspartate) rapidly and reversibly down-regulated AA from quail hypothalamic explants. Addition of the glutamate receptor antagonist NBQX blocked the effect of kainate on AA, confirming the specificity of the treatments (Balthazart et al., 2006). Interestingly, intracellular recording combined with biocytin labeling of aromatase-positive neurons confirm that these neurons are sensitive to glutamate, a finding consistent with the idea that glutamatergic inputs on aromatase cells acutely regulate AA (Cornil et al., 2000, 2004). In parallel, *in vivo* retrodialysis of glutamate in the zebra finch telencephalon induced a significant reduction of local E2 concentration, while NMDA had no apparent effect (Remage-Healey et al., 2008).

### Dopamine

It should be noted that dopaminergic signaling could also be involved in the rapid modulation of AA. Double immunocytochemistry highlighted a close interaction between dopaminergic fibers (tyrosine hydroxylase-positive structures) and aromatase-positive cells in the hypothalamus of Japanese quail (Balthazart et al., 1998). More importantly, several dopaminergic drugs rapidly inhibited AA in homogenates and *in vitro* explants obtained from the preoptic area hypothalamus of male Japanese quail (Baillien and Balthazart, 1997; Balthazart et al., 2002). This inhibition was fully reversible in explant when the compounds were removed from the incubation medium (Absil et al., 2001). Surprisingly, agonists as well as antagonists of dopamine, specific for the D1 or D2 receptors, depressed AA in explants incubated *in vitro*, raising questions about the exact mechanism underlying the potential implication of D1 or D2 receptors. In addition, the observation that the dopamine reuptake inhibitor nomifensine had no effect in homogenates but strongly inhibits AA in explants suggests that the accumulation of dopamine within the synaptic cleft is the cause of AA inhibition.

### GABA

Another neurotransmitter, the inhibitory amino acid GABA, did not affect the enzymatic activity in quail preoptic hypothalamus explants nor *in vivo* in zebra finch telencephalon (Balthazart et al., 2001b; Remage-Healey et al., 2008). It is therefore obvious that the direct links between the activation of the membrane receptor, calcium release and the exact intracellular pathway(s) involved in the rapid modulation of AA require further investigation.

## Post-translational modifications of aromatase activity by phosphorylation

Post-translational modifications are a common mechanism involved in the control of protein activity and is a widely used regulatory mechanism in the brain to control for example NMDA receptor activity in hippocampal neurons (Chen and Roche, 2007) and tyrosine hydroxylase activity, the rate-limiting enzyme in catecholamine synthesis (Albert et al., 1984; Daubner et al., 1992). These phosphorylations are catalyzed by specific kinases that critically depend on the intracellular concentration of Ca^++^ and Mg^++^. Interestingly, previous studies have implicated divalent cations in the control of AA (Ca^++^: Onagbesan and Peddie, 1989; Mg^++^: Steimer and Hutchison, 1991). We therefore wondered whether Ca^++^ and Mg^++^, in addition of adenosine triphosphate ATP as a phosphate donor, during phosphorylation process could also potentially affect AA. We showed that exposure to ATP (2–8 mM), Ca^++^ (0.5–2 mM), and Mg^++^ (2–10 mM) rapidly and significantly inhibited AA in male Japanese quail preoptic area/hypothalamus homogenate (Balthazart et al., 2001b), strongly suggesting that phosphorylations rapidly inhibit this enzymatic activity. To test whether ATP/Mg/Ca conditions induce kinase-dependent protein phosphorylations or whether these conditions result in a non-specific inactivation of AA, we tested effects of the second messengers cAMP and cGMP, respectively activators of protein kinase A, PKA and protein kinase G, PKG. These kinase activators did not modify AA in the absence or presence of ATP/Mg/Ca (Balthazart et al., 2003). We also tested the effects of numerous kinase inhibitors on the AA of preoptic area/hypothalamic homogenates in the presence or absence of ATP/Mg/Ca (Balthazart et al., 2003). Several of these inhibitors significantly blocked the inhibition of AA induced by the addition of ATP/Mg/Ca while others had no effect. The general serine/threonine (Ser/Thr) kinase inhibitor Staurosporine (STAU), and the general tyrosine (Tyr) kinase inhibitor genistein (GEN) significantly blocked the inhibition produced by ATP/Mg/Ca while they had no effect on basal AA. Similar effects (blockade of inhibition) were also observed in presence of the PKC inhibitor bisindolylmaleimide, the myosin light chain kinase inhibitor ML7, the inhibitor of PKA and PKC H7, the specific inhibitor of PKA/PKG H9, the PKA inhibitor H89 and the calmodulin antagonist trifluoperazin. Altogether, these data indicate that rapid modulation of AA results from the phosphorylation of both Tyr and Ser/Thr residues (Balthazart et al., 2001a, 2003). It should be noted that the potential quantitative role of each kinase family in the rapid modulation of AA in homogenates seems to vary according to the sex: While the addition of protein kinase inhibitors blocked the ATP/Mg/Ca-dependent inhibition AA in both sexes, the magnitude of these effects were sexually differentiated and usually more pronounced in females compared to males (Konkle and Balthazart, 2011). Moreover, phosphorylating conditions affected AA differentially in 2 subcellular compartments isolated via differential ultracentrifugation from zebra finch telencephalon. The reduction of AA by phosphorylating conditions was significantly more pronounced in the fraction containing synaptosomes (P2), as compared to microsomes (endoplasmic reticulum, P3 fractions, Cornil et al., 2012b).

We further investigated whether the rapid inhibition of AA by ATP/Mg/Ca-dependent phosphorylation processes is specific to the neuronal environment or can be observed in other aromatase-rich tissues. Effects of phosphorylating conditions were quantified in ovary and ovarian follicles homogenates from Japanese quail and demonstrated a drastic decrease in enzymatic activity within 15 min (Charlier et al., 2011). We also transiently and stably expressed human aromatase in HEK293 cells. Similarly to what was observed in the preoptic area/hypothalamus of Japanese quail and telencephalon of zebra finch, a K^+^-induced depolarization triggered a pronounced but transient inhibition of AA expressed by transfected HEK293 cells. In addition, we also showed that phosphorylating conditions did not affect the enzyme affinity for the substrate but only changed the maximum velocity of reaction. Interestingly, the rapid enzymatic inhibition induced by depolarization also involved the activity of protein kinases as the addition of staurosporine (Ser/Thr kinase inhibitor) or Genistein (Tyr kinase inhibitor) blocked the effect of K^+^-induced depolarizations on AA (Charlier et al., 2011). These observations are contrasting to what is observed in the breast cancer cell line MCF-7. Indeed, Catalano et al. (2009) showed that E2 rapidly modulates c-Src kinase action on aromatase tyrosine residues to enhance AA. It should however be noted that these effects were observed after 5 h of incubation and might be underlying different physiological processes.

In both cases however, the rapid change in AA is reversible, suggesting (1) that phosphorylation does not involve protein degradation when AA is reduced (Charlier et al., 2011) and (2) phosphatase is required to restore the un-phosphorylated stage. Interestingly, the general inhibitor of protein phosphatases (PPase), vanadate, did not significantly increase AA but reduced it in male quail preoptic area/hypothalamic homogenates. In addition, alkaline PPase inhibited AA in a dose-dependent manner in the presence, as well as in the absence, of ATP/Mg/Ca (Balthazart et al., 2005). In contrast, preincubation with acid PPase completely blocked the inhibitory effects of ATP/Mg/Ca on AA although the addition of the PPase was unable to restore AA after the enzymatic activity had been inhibited by the phosphorylating conditions. Moreover, high concentrations of acid PPase also moderately inhibited AA in the absence of ATP/Mg/Ca (Balthazart et al., 2005). It should be noted that recent work on human placental choriocarcinoma cell line JEG-3 demonstrated that microsomal aromatase was rapidly inactivated with calcium, magnesium, and ATP, similarly to what is observed in quail preoptic area/hypothalamus homogenate and transfected cell lines, but the protein was then subsequently degraded in these conditions (Hayashi and Harada, 2014). More specifically, aromatase was protected from protein degradation by treatment with alkaline phosphatase, whereas degradation was enhanced by treatment with the phosphatase (calcineurin) inhibitors FK506 and cyclosporin A. Furthermore, aromatase was protected from degradation by treatment with kinase inhibitors, especially the calcium/calmodulin kinase inhibitors KN62 and KN93 (Hayashi and Harada, 2014).

It is therefore possible aromatase can first be rapidly inactivated by phosphorylation, then follow 2 different paths: either (1) its activity is restituted by dephosphorylation following interaction with phosphatase, or (2) the absence of dephosphorylation leads to subsequent degradation, i.e., a permanent inactivity of the enzyme. However, the specific enzymes involved in the phosphorylation-dephosphorylation mechanisms associated to the rapid and reversible control of AA are not fully defined and are currently under further investigation. It should also be noted that tyrosine phosphatase PTP1B was shown to reduce AA in MCF-7 and ZR75 breast cancer cells (Barone et al., 2012). Altogether, this suggests that some level of phosphorylation is required for a fully functional activity but future work should define in more detail the intricate role of phosphatases and kinases in the control of AA in different cell types.

## Evidence for the direct phosphorylation of the aromatase protein

Our next question was to define whether phosphorylations controlling enzymatic activity directly affect the aromatase itself or modulate the activity of a cofactor that could secondarily regulate AA. We purified by immunoprecipitation the aromatase protein from quail preoptic area/hypothalamic homogenates and the presence of phosphorylated residues on the purified protein was investigated by Western blotting using anti-phosphoserine, anti-phosphothreonine or anti-phosphotyrosine antibodies. Confirming the pharmacological finding described above, phosphorylated Ser, Thr, or Tyr were identified by the specific antibodies at the level of the electrophoresis band corresponding to aromatase and the intensity of the phosphorylation signal was denser when homogenates were pre-incubated with ATP/Mg/Ca as compared to homogenates incubated in control conditions (Balthazart et al., 2003).

Similar experiments were performed on engineered human aromatase containing a c-myc tag that allows its specific immunoprecipitation. HEK293 cells transfected with this construct were incubated with [γ−^32^P]-ATP in phosphorylating or non-phosphorylating (control) conditions. The radioactive aromatase c-myc in phosphorylating conditions was clearly observed at the expected molecular weight while only a low-intensity band was present in control conditions (Charlier et al., 2011). In additional experiments, the immunoprecipitated aromatase obtained after a 5 min-incubation of the cell lysate in phosphorylating conditions was clearly visible at the expected size on immunoblot when visualized with anti-phosphoserine antibody, while that band was not present in different control conditions (Charlier et al., 2011). This set of experiments clearly supports the hypothesis that the aromatase protein itself is rapidly phosphorylated in the presence of ATP/Mg/Ca, leading to a reduction of its enzymatic activity. Similarly, immunoprecipitation of His6-tagged aromatase in MCF-7 cells and investigation of phosphorylated amino acid by immunoblotting revealed that aromatase tyrosine residue is directly targeted by the kinase c-Src (Catalano et al., 2009, 2014).

## Identification of aromatase residues involved in the rapid control of activity

This set of pharmacological experiments on quail hypothalamus explants and homogenates and the manipulations performed with immunoprecipitated human aromatase indicated that the inhibition of AA by phosphorylation is likely to be catalyzed by the activity of two Ser/Thr kinases, protein kinase A (PKA) and C (PKC), although the implication of tyrosine kinase and other Ser/Thr kinases cannot be completely ruled out (Balthazart et al., 2003; Charlier et al., 2011). The quail and human aromatase protein sequence were analyzed using bioinformatic tools to identify the potential phosphorylation sites, highly conserved amongst several avian and mammalian species. Fifteen and nineteen putative phosphorylation sites were identified for quail and human sequences respectively using the open access NETPHOS 2.0 program. These sites included 10 of the 27 serines, 5 of the 24 threonines, and 4 of the 17 tyrosines residues on the human sequence and we paralleled these findings with the results obtained using NETPHOSK, highlighting consensus sequences corresponding to PKC and PKA, protein kinases shown to affect quail and human AA during the pharmacological experiments (Balthazart et al., 2003; Charlier et al., 2011). The output pointed to 6 different residues, five serines (S118, S167, S247, S267, S497), and one threonine (T143) on the human sequence. It should be pointed out that other kinases might also be involved, as consensus binding sequence were observed on both quail and human aromatase. These were however not investigated. From this analysis, we decided to focus our attention on 6 different residues: S247, S267, and S497 that had high scores in both the predictive phosphorylation sites and PKA or PKC recognition consensus sequence; T462 and T493 that correspond to positions S455 and S486 in quail aromatase, two 2 residues that were predicted to be involved in the phosphorylation of aromatase (Balthazart et al., 2003). In addition, *in vitro* studies of the mouse aromatase performed by 2 different groups suggested that 2 other amino acid residues, namely serine S118 (Miller et al., 2008) or tyrosine Y361 (Catalano et al., 2009) can be phosphorylated. Indeed, the phosphorylation of Y361 triggered by the tyrosine-kinase c-Src seems involved in the E2-dependent upregulation of AA in breast cancer cell lines (Catalano et al., 2009) while phosphorylation of S188 by AGC-like kinases, including PKC and PKA, seems important for the stabilization of the protein, and therefore for the expression of AA (Miller et al., 2008). It should be noted that the effects of the phosphorylation status of these 2 residues on the rapid change of AA was not investigated and we explored the potential implication of S188 phosphorylation (not Y361) in the rapid control of AA.

Altogether, we investigated the consequence of mutating 6 S/T residues into alanine (A) using the human aromatase as template. All mutants expressed normal baseline AA and pre-incubation with ATP/Ca/Mg for 15 min markedly reduced this enzymatic activity in the 6 different mutants alone (S118A, S247A, S267A, T462A, T493A, S497A) or in combination (S267A-S247A, T493A-T462A-S267A, T493A-T462A-S267A-S247A, S497A-T493A-T462A-S267A-S247A) roughly to the same extent as in wild type enzyme, clearly showing that these single or combined mutations did not block the rapid inhibition of aromatase by phosphorylating conditions. The absence of effect, against all expectations, was thoroughly discussed in the original article (Charlier et al., 2011). In summary, it is likely that a combination of several phosphorylated residues that was not tested here is required to control AA. The control by multiple kinases reinforces the idea that several amino acids must concomitantly be phosphorylated to modify AA. It is possible that other consensus sites for phosphorylations and for other types of kinases that were also predicted on the quail and human aromatase sequences might be involved. Alternatively, it is possible that the control of AA by phosphorylations depends on the modulation of certain factors interacting with aromatase, in addition to the enzyme itself. Aromatase requires the presence of an active NADPH-cytochrome P450 reductase for electron transfer (Thompson and Siiteri, 1974; Miller, 2005; Hong et al., 2009). The phosphorylation of the reductase could be a control mechanism affecting the electron transfer and this idea should be further investigated.

## Behavior and rapid modulation of aromatase activity

It is important to note that the change in enzymatic activity resulting from the phosphorylation of aromatase is physiologically and behaviorally relevant. Indeed, rapid changes in AA were demonstrated to occur *in vivo* in response to social interactions in a few species investigated to date, including birds and fish. For example, expression of appetitive or consummatory sexual behavior rapidly reduces AA in specific brain regions in the preoptic-hypothalamic area of quail after 5–30 min of sexual interaction with a receptive female (Cornil et al., 2005a; de Bournonville et al., 2013) while exposure to acute restraint stress, or stress-related hormones such as AVT and corticosterone lead to an increase in AA in both male and female quail (Dickens et al., 2011, 2013). Male removal from a socially stable group of the sex-changing fish *Lythrypnus dalli* resulted in rapid increases in aggression associated with a rapid reduction in brain AA in the dominant female (Black et al., 2005, 2011). Conversely in zebra finches, a brief exposure to song (30 min) resulted in an increase of AA in NCM within the telencephalon (Remage-Healey et al., 2009). While behavior can affect AA, the reverse is also true and recent studies showed that rapid changes in AA modulate behavior and sensory processing. Indeed, an acute systemic or central administration of aromatase inhibitors such as Vorozole or Androstatrienedione significantly and rapidly reduced male sexual behavior in quail and mice (Cornil et al., 2005b; Taziaux et al., 2007; Seredynski et al., 2013), approach behavior toward females in male goldfish (Lord et al., 2009), aggressive behavior in Beach and California mice (Trainor et al., 2007, 2008) and vocalizations in midshipman fish (Remage-Healey and Bass, 2004). Fadrozole, another aromatase inhibitor, rapidly suppressed the preference for a bird's own song in male zebra finches (Remage-Healey et al., 2010) and reduced song-elicited neuronal firing rate in zebra finches and prevented the expression of hearing-driven gene expression in NCM, a brain regions involved in song discrimination in songbirds (Tremere et al., 2009). Altogether, this clearly confirms that the rapid modulation of AA, likely by phosphorylation, is of physiological importance. This also shows that the rapid inhibition of AA rapidly influences the physiology and behavior.

## Catabolism of estrogens

Therefore, there must exist some mechanisms that are able to rapidly clear locally-produced estrogens to terminate their effects. The half-life of E2 in the brain is not known, although calculations derived from pharmacokinetic data estimated that its half-life in the blood is below 30 min (Johnson and van Tienhoven, 1981; Tsang and Grunder, 1984). The liver is known to express several enzymes involved in the clearance of circulating estrogens, notably CYP1A1, CYP1A2, CYP1B1, and CYP3A (Whirl-Carrillo et al., 2012). These enzymes catalyze the conversion of estrogen into inactive (or less active) water-soluble metabolites by oxidative metabolism and allow for further sulfation by sulfotransferases, glucuronidation by glucoronyltransferases, or methylation by catecholmethyltransferases. The elimination of plasma estrogens from the organism is ensured through excretion following their conjugation. Although this type of degradation of estrogens occurs mainly in the liver, some catabolic activity is also observed in the brain, albeit to a much lower extent. We hypothesized that the rapid elimination of estrogens in the brain involves the enzyme aromatase itself. Indeed, a few studies suggest that, in addition to its estrogen synthase activity, aromatase also catalyzes estrogen hydroxylation. A previous study in Japanese quail showed that 2-hydroxylase enzymatic activity is present in all brain regions that are known to contain a high level of AA, including the median preoptic nucleus and the ventromedial nucleus of the hypothalamus (Balthazart et al., 1994), suggesting that the same protein is involved in both the production and the conversion of estrogens. However, no sex difference in 2-hydroxylase activity was identified here, while AA is higher in males than in females in almost every brain region. The first evidence that aromatase protein also had estrogen hydroxylase activity came from Osawa et al. (1993). They demonstrated that purified aromatase obtained from placental microsomes demonstrates an estrogen-2-hydroxylase activity in certain conditions. Both aromatase and estradiol 2-hydroxylase activities were observed after purification of human placental microsomal cytochrome P-450 by monoclonal antibody-based immunoaffinity chromatography and gradient elution. Moreover, they confirmed the concomitant existence of estradiol hydroxylase activity with AA by expressing human aromatase in CHO cells. Not only did the purified aromatase exhibit the hydroxylase activity but testosterone and androstenedione competitively inhibited estradiol 2-hydroxylation, and conversely, estrone and estradiol competitively inhibited aromatization of both testosterone and androstenedione. Similarly, equine aromatase expressed *in vitro* has a clear hydroxylase activity (Almadhidi et al., 1996). A specific antiserum raised against purified testicular equine P450arom and known to inhibit AA was also found to inhibit the estrogen hydroxylase activity of equine placental microsomes. Furthermore, the estrogen hydroxylase activity was inhibited in a dose-dependent manner by different aromatase inhibitors, including fadrozole and 4-hydroxyandrostenedione (Almadhidi et al., 1996). It remains to be determined whether the phosphorylation of the same residue(s) leads to the rapid reduction of both estrogen synthase and estrogen hydroxylase activity.

## Conclusions

**Figure 1:**
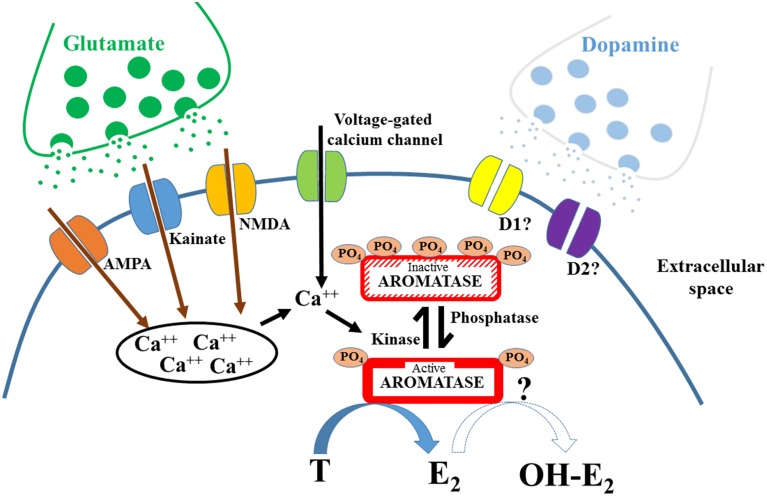
**Schematic diagram representing the mechanisms involved in the rapid control of aromatase activity**. Phosphorylations (PO_4_) rapidly modulate aromatase activity, inhibiting the transformation of testosterone (T) into 17β-estradiol (E2). It is likely that these modifications are induced by calcium-voltage channels, by glutamatergic receptors and/or by dopaminergic receptors. The increase of intracellular calcium (Ca^++^), either from intracellular storage or from the activation of voltage-gated channel is in most cases a prerequisite for the inhibition of aromatase activity. Change in phosphorylation level could also affect the hydroxylase activity of aromatase.

Altogether, a few laboratories have accumulated strong evidence showing that AA can be rapidly modulated via post-translational modifications, most notably via phosphorylation (see Figure 1 for general model). The rapid modulation of AA by phosphorylating conditions is a widespread mechanism present in different tissues affecting aromatase from various species, including humans. The mechanisms leading to these modifications remain poorly understood, but it is increasingly clear that the enzymatic changes must result in a local rapid modulation of estrogens availability and consequently in a modification of cellular estrogen-dependent events that are not mediated by the genomic actions of these steroids. The phosphorylation/dephosphorylation processes provide a new widespread mechanism by which estrogens concentration could be rapidly altered in the brain and other tissues, albeit likely in different directions. It would be interesting to test whether precise phosphorylation patterns affect one type of enzymatic activity while leaving the other untouched or whether phosphorylation is a general shut-down switch for all enzymatic activity displayed by the aromatase protein.

### Conflict of interest statement

The authors declare that the research was conducted in the absence of any commercial or financial relationships that could be construed as a potential conflict of interest.
